# Novel structural features in two ZHX homeodomains derived from a systematic study of single and multiple domains

**DOI:** 10.1186/1472-6807-10-13

**Published:** 2010-05-28

**Authors:** Louise E Bird, Jingshan Ren, Joanne E Nettleship, Gert E Folkers, Raymond J Owens, David K Stammers

**Affiliations:** 1 Oxford Protein Production Facility, Division of Structural Biology, Henry Wellcome Building for Genomic Medicine, University of Oxford, Roosevelt Drive, Headington, Oxford, OX3 7BN, UK; 2 Bijvoet Centre for Biomolecular Research, University of Utrecht, Netherlands; 3 Division of Structural Biology, Henry Wellcome Building for Genomic Medicine, University of Oxford, Roosevelt Drive, Headington, Oxford, OX3 7BN, UK

## Abstract

**Background:**

*Zhx*1 to 3 (zinc-fingers and homeoboxes) form a set of paralogous genes encoding multi-domain proteins. ZHX proteins consist of two zinc fingers followed by five homeodomains. ZHXs have biological roles in cell cycle control by acting as co-repressors of the transcriptional regulator Nuclear Factor Y. As part of a structural genomics project we have expressed single and multi-domain fragments of the different human ZHX genes for use in structure determination.

**Results:**

A total of 30 single and multiple domain ZHX1-3 constructs selected from bioinformatics protocols were screened for soluble expression in *E. coli *using high throughput methodologies. Two homeodomains were crystallized leading to structures for ZHX1 HD4 and ZHX2 HD2. ZHX1 HD4, although closest matched to homeodomains from 'homez' and 'engrailed', showed structural differences, notably an additional C-terminal helix (helix V) which wrapped over helix I thereby making extensive contacts. Although ZHX2 HD2-3 was successfully expressed and purified, proteolysis occurred during crystallization yielding crystals of just HD2. The structure of ZHX2 HD2 showed an unusual open conformation with helix I undergoing 'domain-swapping' to form a homodimer.

**Conclusions:**

Although multiple-domain constructs of ZHX1 selected by bioinformatics studies could be expressed solubly, only single homeodomains yielded crystals. The crystal structure of ZHX1 HD4 showed additional hydrophobic interactions relative to many known homeodomains via extensive contacts formed by the novel C-terminal helix V with, in particular, helix I. Additionally, the replacement of some charged covariant residues (which are commonly observed to form salt bridges in non-homeotherms such as the *Drosophila *'engrailed' homeodomain), by apolar residues further increases hydrophobic contacts within ZHX1 HD4, and potentially stability, relative to engrailed homeodomain. ZHX1 HD4 helix V points away from the normally observed DNA major groove binding site on homeodomains and thus would not obstruct the putative binding of nucleic acid. In contrast, for ZHX2 HD2 the observed altered conformation involving rearrangement of helix I, relative to the canonical homeodomain fold, disrupts the normal DNA binding site, although protein-protein binding is possible as observed in homodimer formation.

## Background

The gene for ZHX1 (zinc-fingers and homeoboxes 1) was originally cloned by immuno-screening using a monoclonal antibody raised against the endothelial adipose stromal cell line 14F1.1 [1]. ZHX1 was independently identified through studies which demonstrated its interaction with the activation domain of NF-YA, [2,3]. NF-YA is a component of nuclear factor Y (NF-Y), a trimeric transcriptional activator. NF-Y recognizes the regulatory CCAAT element in the proximal and distal enhancer regions of many genes in either orientation, resulting in upregulation [4]. NF-Y plays a pivotal role in regulating cell cycle factors such as cyclin A & cdc2.

Further members of the gene family, ZHX2 and ZHX3, were identified through their ability to form heterodimers with ZHX1 [5,6]. ZHX1 to 3 are all members of the zinc-fingers sub-family of the homeodomain superfamily [7,8]. ZHXs are multidomain proteins comprising two C_2_H_2 _zinc finger motifs and five homeodomains [1,5,9]. Both homeodomains and zinc fingers are short protein modules involved in protein-DNA and/or protein-protein interactions; they are frequently associated with roles in transcriptional regulation.

All members of the ZHX family are reported to be able to form both homo- and heterodimers via the region containing homeodomain 1 [6,9-12]. More recently, ZHX1 has also been reported to interact with the bifunctional transcription factor BS69. Transcriptional activation by BS69 appears to be suppressed by ZHX1 [13].

ZHXs are ubiquitously expressed in adults with variations in levels observed between tissues [5,9,10]. Biological roles for the ZHX protein family are beginning to emerge from various studies. In transient co-transfection assays ZHX isoforms have all been shown to function as transcriptional repressors [5,6,11]. Consistent with this designation of function, retroviral insertion into the *Zhx2 *locus in BALB/cJ mice causes, probably in a gene-dosage-dependent manner, the hereditary persistence of α-fetoprotein and *H*19 expression in the liver [14]. ZHX proteins have been shown to regulate podocyte gene expression. Thus ZHX3 mediates changes in localization during the pathogenesis of glomerular disease [15]. It was shown that the level of hetero-dimerisation of ZHX3 with ZHX1 or ZHX2 disfavoured the nuclear localization of ZHX3. Loss of such heterodimer formation leads to nuclear uptake of ZHX3 associated with the disease state [16].

The division of multidomain proteins into fragments and use of high throughput approaches to aid structure determination in structural genomics projects have been previously documented [17,18]. We have applied these methods to the structural analysis of the highly modular multi-domain ZHX family, where in each case the complete gene failed to express in bacterial systems. Soluble proteins were purified and used to set up crystallization trials, leading to structure determinations of both ZHX1 HD4 and ZHX2 HD2. Whilst many 3-D structures of homeodomains have been determined by crystallography and NMR [7], we report two ZHX homeodomain structures which contain novel features. Additionally, the expression work undertaken here also lead to the structure determination of the double zinc finger domain by NMR (PDB code 2GHF) [19].

## Methods

### Bioinformatics

**Figure 1:**
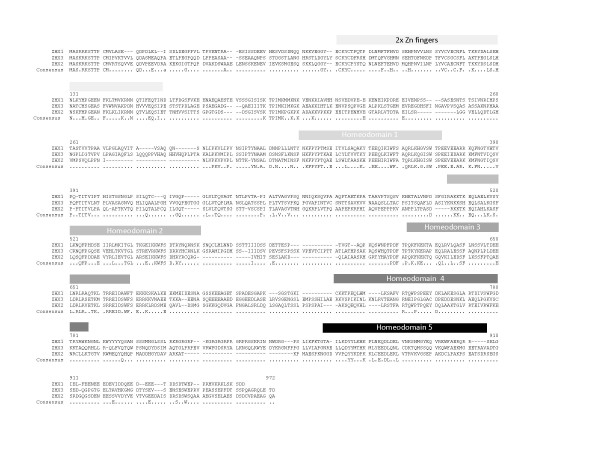
**Alignment of amino acid sequences for human ZHX paralogs**. Positions of zinc finger and homeodomains derived from the alignment are indicated.

Domain boundaries for ZHX proteins used in designing truncations were derived from a combination of amino acid sequence alignments (Figure 1) together with domain designations shown in the NCBI protein database. Additionally, low complexity regions as predicted by PONDR http://www.pondr.com and RONN [20] were eliminated from the constructs.

### Cloning of ZHX E. coli expression constructs

All ZHX constructs were derived using ligation-independent cloning methods by either Gateway (Invitrogen) or Infusion (Clontech/BD Biosciences) technologies [21], [22]. Expression vectors were made using high-throughput parallelized protocols in a 96-well plate format. Descriptions of all constructs together with the PCR primers utilized are shown in Table 1.

**Table 1:** Expression constructs for human ZHXs

Protein	Construct OPPF_	Domain/s	aa range	5' primer	3' primer	Vector	Tag
ZHX1	1610	Full-length	1-874	ATGGCAAGCAGGCGAAAATCAAC	TCAGTCATCTGATTTAGACAGCTTCCG	pDEST14	N-His
	1611	HD1	284-343	AATAGCATTCCCACCTACAATGCTGC	CCAACTAACACCATGTTTTAAACGTTGGGC	pDEST14	N-His
	1612	HD3	556-633	CAGCCTAAGCAATCCTGGAATCC	ATCTATTTCCATTTTCTCTTCCTTTAAAGCTTTTG	pDEST14	N-His
	1613	HD4	655-731	GCACCTAAGTCAGGGAGTACAGGCAAG	TGAATTGGCGCTCTGATAGTAGTAGTACCATTTC	pDEST14	N-His
	1614	HD5	770-824	GACAGGGGACCATCACTCATAAAATTTAAAAC	TCTTCTCTGTCTTTCTGCAAACCACTCTCTG	pDEST14	N-His
	1615	HD3-4	556-731	CAGCCTAAGCAATCCTGGAATCC	TGAATTGGCGCTCTGATAGTAGTAGTACCATTTC	pDEST14	N-His
	1616	HD1-5	284-824	AATAGCATTCCCACCTACAATGCTGC	TCTTCTCTGTCTTTCTGCAAACCACTCTCTG	pDEST14	N-His
	1632	2ZF	60-153	AATCAGCAAAATAAAAAAGTTGAAGG	AAAAGTCAGATCATTTATTGTTTGTTC	pDEST14	N-His
	1633	2ZF	55-156	TCTGTGGATTCAGACAATCAGC	ACTACCATCAAAAGTCAGATCATTTATTG	pDEST14	N-His
	1634	HD2	464-523	TCATTTGGCATTCGGGCAAAAAAGAC	CTTTGAATTTCTCTGGTTGTACCTTGTG	pDEST14	N-His
	1635	HD2-3	464-633	TCATTTGGCATTCGGGCAAAAAAGAC	ATCTATTTCCATTTTCTCTTCCTTTAAAGCTTTTG	pDEST14	N-His
	1636	HD2-4	464-731	TCATTTGGCATTCGGGCAAAAAAGAC	TGAATTGGCGCTCTGATAGTAGTAGTACC	pDEST14	N-His
	1637	HD2-5	464-824	TCATTTGGCATTCGGGCAAAAAAGAC	TCTTCTCTGTCTTTCTGCAAACCACTCTCTG	pDEST14	N-His
	2264	HD3	556-633	CAGCCTAAGCAATCCTGGAATCC	ATCTATTTCCATTTTCTCTTCCTTTAAAGCTTTTG	pOPINF	N-His -3C
	2265	HD4	655-731	TCTGTGGATTCAGACAATCAGC	TTCTATACCTAATTCTGATCTTCTCTGTC	pOPINF	N-His -3C
	2465	All	55-830	TCTGTGGATTCAGACAATCAGC	TTCTATACCTAATTCTGATCTTCTCTGTC	pOPINF	N-His -3C
ZHX2	2268	2ZF	60-158	GTGATAGAGGTGAAATCTATGGG	GGTTTCGATGGACTGTTCCAAG	pDEST14	N-His
	2269	HD1	267-321	TACAACTCTGCCCTGGATACAA	CCAGCTGATGCCATGCTTTAAG	pDEST14	N-His
	2270	HD2	444-500	CGCAAGAAGACAAAGGAGCAG	GTGGACGATGCCCCTTTGAC	pDEST14	N-His
	2271	HD3	530-582	CCCCAGAAGTTCAAAGAGAAAAC	CTTCCGCCTCTCCGAGAACC	pDEST14	N-His
							
	2272	HD4	635-686	AAAAGTCAAGAACAGGTTCATCTC	TCCCGTTTTCAGCAAGCATCTG	pDEST14	N-His
	2273	HD2-3	444-582	CGCAAGAAGACAAAGGAGCAGA	CTTCCGCCTCTCCGAGAACC	pDEST14	N-His
	2466	All	55-745	TCCAAAGAAAACGAAGTGATAGAGGTGAAATC	CAACTTCTCCAAGTCCTCTTCGCAG	pOPINF	N-His -3C
ZHX3	2274	2ZF	66-153	AATGGGCATCGGAGCACTTTAG	CTGCTCCACAACCACATGATTG	pDEST14	N-His
	2275	HD1	306-363	ATTCCAACGTACAATGCAGCC	CCAGCTGATCCCCTGCTTCAG	pDEST14	N-His
	2276	HD2	500-553	AAGAAATCTCATGAACAGCTGTCAG	CTTCAAGTTCCGGCAGTGGTATC	pDEST14	N-His
	2277	HD3	621-664	CCTGAGCAGCTCAGAGCC	CCGTCTCTCTGAAAACCAGC	pDEST14	N-His
	2278	HD4	758-826	AAACTGGCAGAGCAGCTCC	CCATTTGAGTTGGCCGTTCTTC	pDEST14	N-His
	2279	HD2-3	500-664	AAGAAATCTCATGAACAGCTGTCAG	CCGTCTCTCTGAAAACCAGC	pDEST14	N-His
	2467	All	61-966	GGCTCTACACTGGCCAATGGGC	AGGTAAAGGAAAGGTCCTACATTGTGGG	pOPINF	N-His -3C

For the primers only the regions homologous to the target sequence are shown. 5' primers had GGGGACAAGTTTGTAC

CCAAAAAAGCAGGCTTCGAAGGAGATAGAACCATGGCACATCACCACCACCATCAC and AAGTTCTGTTTCAGGGCCCG 5' extensions, while 3' primers had GGGG

ACCACTTTGTACAAGAAAGCTGGGTCTCA and ATGGTCTAGAAAGCTTTA 5' extensions for cloning into pDEST14 or pOPINF vectors using either Gateway technology or the Infusion cloning system respectively.

PCR was performed in a 50 μl reaction mix using KOD Hi-Fi polymerase according to the manufacturer's instructions (Novagen) with 30 pmol of each forward and reverse primers, and 1 μl of plasmid DNA containing human ZHX genes (approximately 100 ng/μl) as template per reaction.

All constructs, with the exception of OPPF_2264, OPPF_2265, and OPPF_ 2465-67, were cloned into the pDEST14 vector using Gateway technology according to the manufacturer's protocols. A Shine-Dalgarno sequence together with an N-terminal hexa-histidine tag were incorporated into the 5' primer in order to give rise to protein products that did not have translated *att *sites. The remaining PCR products were cloned into pOPINF using the In-Fusion™ cloning system in the In-Fusion™ Dry-Down 96-well plate format according to the manufacturers protocol (Clontech/BD Biosciences). These latter products have an N-terminal hexa-histidine tag together with a rhinovirus 3C protease cleavage site. The Infusion methodology and pOPINF vector have been previously described in detail [22].

### Expression Screening

A 1 μl aliquot of each expression construct was transformed into B834 (DE3) pRARE and Rosetta pLysS *E. coli *(Novagen), respectively, in a 96-tube format. Transformants were selected by plating on 24-well culture plates containing 1 ml of LB Agar/well, supplemented with the 50 μg/ml carbenicillin, 35 μg/ml chloramphenicol. Incubation was continued for 18 hours at 37°C before individual colonies were used to inoculate, 500 μl GS96 (Bio101, QBioGene, Cambridge, U.K.) supplemented with 0.05% v/v glycerol, 1% w/v glucose, 50 μg/ml carbenicillin and 35 μg/ml chloramphenicol, in 96-position deep-well plates. The plates were covered with gas-permeable adhesive seals and shaken at 225 rpm at 37°C for 18 hours. 50 μl of each overnight culture was then used to inoculate (in four 24-well deep-well plates) either 2.5 ml of GS96 supplemented with 0.05% v/v glycerol or with 2.5 ml of GS96 supplemented with 0.05% v/v glycerol and Overnight Express additives (Novagen). 50 μg/ml carbenicillin and 35 μg/ml chloramphenicol were present in both sets of culture media. The diluted cultures were grown at 37°C, shaken at 225 rpm for 3 hours before the temperature was reduced to 20°C. ITPG to a final concentration of 0.5 mM was then added to the media (not supplemented with Overnight Express) with shaking for a further 18 hours at 20°C.

A 1.5 ml aliquot of each culture was then transferred to a 2 ml 96-deep-well plate using a Theonyx robot (Aviso Gmbh, Gera, Germany) and harvested by centrifugation at 6000*g *for 10 minutes at 4°C before removal of waste media. Pelleted cells were frozen at -80°C for at least 30 minutes prior to screening for soluble His_6_-tag protein expression using either the Theonyx or BR8000 robotic platforms with standard QIAgen Ni-NTA magnetic bead protocols. Purified proteins eluted from the Ni-NTA beads were analysed on SDS-Page gels (Criterion™ 10-20% gradient gels-Biorad.), visualised with SafeStain™ (InVitrogen) and the level of soluble expression scored (Table 2).

**Table 2:** Small-scale expression analysis of ZHX constructs

Protein	Construct OPPF_	Domain/s	Expression
ZHX1	1610	Full-length	Expression
	1611	HD1	0
	1612	HD3	+
	1613	HD4	++
	1614	HD5	++
	1615	HD3-4	0
	1616	HD1-5	++
	1632	2ZF	NA
	1633	2ZF	++
	1634	HD2	++
	1635	HD2-3	0
	1636	HD2-4	+++
	1637	HD2-5	+++
	2264	HD3	NA
	2265	HD4	++
	2465	All	++
ZHX2	2268	2ZF	0
	2269	HD1	+
	2270	HD2	0
	2271	HD3	NA
	2272	HD4	NA
	2273	HD2-3	0
	2466	All	++
ZHX3	2274	2ZF	0
	2275	HD1	+
	2276	HD2	0
	2277	HD3	0
	2278	HD4	0
	2279	HD2-3	0
	2467	All	+

NA: expression vector not available. The extent of soluble expression is shown on a scale of + to +++, whilst 0 indicates no expression.

### Expression and purification of recombinant protein

All constructs were transformed into Rosetta pLysS (Novagen) colonies then placed on agar plates supplemented with 50 μg/ml carbenicillin and 35 μg/ml chloramphenicol. Colonies from the plates were used to inoculate 2 l of GS96 supplemented with Overnight Express additives (Novagen), 0.05% v/v glycerol, 50 μg/ml carbenicillin and 35 μg/ml chloramphenicol, the media were distributed between three 2 l baffled flasks. The flasks were shaken at 240 rpm at 37°C for 6 hours then cooled to 20°C for a further 22 hours. The cells were harvested by centrifugation, resuspended in 50 ml of 50 mM Tris pH 7.5, 20 mM imidazole, 500 mM NaCl (bufferA) and, if not used immediately, were stored frozen at -80°C. The thawed supernatant had 50 μl of 40,000 units/ml DNAseI (Sigma) and three Complete™ mini EDTA free protease inhibitor tablets (Roche Diagnostics Ltd.) added. Cell suspensions were lysed using a Basic-Z Cell Disruptor (Constant Systems) at 30 Kpsi; the supernatant was clarified by centrifugation at 48000 g for 15 minutes. All proteins were purified using His-Affinity chromatography on an Äkta-FPLC (GE Healthcare) as a first step. Briefly, after loading the clear lysate onto a 1 ml nickel-charged HiTrap Chelating Sepharose FF column (GE Healthcare), the protein was washed with buffer A. The proteins were step-eluted with buffer B (50 mM Tris, pH 7.5, 500 mM NaCl, 500 mM imidazole). For constructs OPPF_2264 and OPPF_2265 the column was washed with 20 mM Tris pH 7.5, 200 mM NaCl, 0.5 mM DTT (buffer C). One ml of buffer C containing 400 μg GST-rhinovirus 3C protease fusion protein was then injected onto the column which was incubated at 4°C overnight. The column was then connected in series with a 1 ml GST-Trap pre-equilibrated in buffer A (GE Healthcare) to remove the GST-3C protease. The cleaved protein was then eluted at a flow rate of 0.2 ml/minute. The columns were subsequently washed with 10% buffer B (to ensure removal any non-specifically bound cleaved protein). At this point the GST-Trap column was removed, and finally, the Ni-affinity column was washed with 100% buffer B. The purest peak fractions (as determined by SDS/PAGE gel analysis) were pooled. All proteins were applied onto a 16/60 HiLoad Superdex 75 column (GE Healthcare) pre-equilibrated in buffer C + 0.5 mM DTT. Protein containing fractions were analysed on SDS/PAGE gels and pooled appropriately, with yields as shown in Table 3. Single ZHX domain preps were >95% pure, whereas multi-domain proteins showed lower purity. Purified proteins were placed in Vivaspin 6 concentrators (5,000 Da molecular weight cut off, Vivascience) and taken to the maximum protein concentration possible. Concentrated proteins were snap-frozen in liquid nitrogen as small aliquots and stored at -80°C. The molecular weights of all purified proteins were confirmed by LC-ESI-MS.

**Table 3:** Scaled up ZHX homeodomain purification yields and crystallization trials

Protein	Construct OPPF_	Yield/(mg/l)	Number of 96 well plates	Structure
ZHX1	1612	4.5	16	
	1613	2.1	10	
	1615	2.3	14	
	1632	1.8	12	
	1633	2.3	6	
	1635	3.5	17	
	1636	5.9	11	
	2264	4.1	12	
	2265	3.9	32	Yes
ZHX2	2268	11.5	25	
	2273	5.0	14	Yes
ZHX3	2274	2.5	8	
	2279	6.3	8	

An additional construct containing the double ZHX1 zinc-finger domain was expressed & protein purified leading to a three-dimensional structure determined using NMR [19].

### Crystallization, Data Collection and processing

Crystallization trials were carried out by sitting drop-vapor diffusion methods with a 200 nl droplet size using previously reported robotic technologies, protocols and screening kits [23]. Crystals of ZHX domains were obtained under the following conditions:

1) ZHX1 HD4 (OPPF_2265): Hampton NATRIX (2), 2.5 M Ammonium Sulphate, 10 mM Magnesium acetate, MES pH5.6. Optimisation was via an additive screen based on the above condition; 20 mM ATP and 20 mM Phenol were the most effective additives. For crystal cryoprotection during data collection, glycerol (20%,v/v) was added to the well solution.

2) ZHX2 HD2-3 (OPPF_2273): Molecular Dimensions PACTPremier (33), 20% PEG6000, 0.2 M LiCl, HEPES pH7. Optimization of the above gave crystals with the following additives, 0.06 M glycyl-glycyl-glycine, 0.02 M sarcosine or 6% ethylene glycol. The solvent content is 49% for ZHX1 HD4 crystals, assuming two molecules in the asymmetric unit, and 52% for ZHX2 HD2-3 crystals, assuming one molecule per asymmetric unit. It was later shown, during the course of structure determination, that two molecules of HD2 were present, *v.i.*

X-ray data from a single crystal of ZHX1 HD4 were collected in house using a Rigaku generator and MAR345 image plate; 305 images of 1.0° oscillation were recorded. A total of 271 images of 1.0° oscillation from a ZHX2 HD2-3 crystal were collected at beamline BM14, the ESRF (Grenoble, France) operating at a wavelength of 0.976 Å using a MARMOSAIC225 CCD detector. In both cases, crystals were frozen and maintained at 100 K during data collection under a cryostream of nitrogen gas. Indexing and integration of data images were carried out using DENZO, and data were merged with SCALEPACK [24]. Whilst both ZHX1 HD4 and ZHX2 HD2-3 crystals belong to the same space group (*C*2), they have differing unit cell dimensions as shown in Table 4. The statistics for the x-ray data collections are also given in Table 4.

**Table 4:** X-ray data collection and refinement statistics


Data collection details		
Data set	ZHX1 HD4 (OPPF_2265)	ZHX2 HD2 (derived from OPPF_2273)
X-ray source	In House	ESRF-BM14
Wavelength (Å)	1.541	0.976
Space group	*C*2	*C*2
Unit cell (a, b, c [Å])	64.90, 48.84, 49.32, β = 95.2°	96.71, 60.67, 27.71, β = 95.3°
Resolution range (Å)	30.0 - 2.60(2.69-2.60)	30.0 - 2.70(2.80-2.70)
Unique reflections	4674(453)	4459(449)
Completeness (%)	99.7(99.1)	100(100)
Redundancy	8.3(6.5)	5.6(5.6)
Average *I/σI)*	24.3(5.5)	15.4(3.1)
Rmerge	0.102(0.339)	0.111(0.451)
Refinement statistics:		
Resolution range (Å)	30.0 - 2.60	30.0-2.70
No. of reflections(working/test)	4459/214	4214/240
R-factor (R_work_/R_free_)	0.197/0.240	0.203/0.267
No. of atoms (protein/water)	1172/44	938/24
rms bond length deviation (Å)	0.006	0.006
rms bond angle deviation (°)	0.7	0.9
Mean B-factor (protein/water[Å^2^])	44/43	42/38

### Structure determination, refinement and analysis

Molecular replacement used the MOLREP program [25]with related homeodomain structures available from the PDB as search models, as indicated in the Results section.

Models were first refined against all X-ray data in CNS [26] using simulated annealing and positional refinement with main chain NCS restraints followed by individual isotropic *B *factor refinement. Phase modification and averaging combined with automated model building were then carried out with RESOLVE [27]. Cycles of model rebuilding were carried out using the program O [28] and alternated with refinement using CNS and REFMAC [29] resulting in the current models which have R-factors of 0.197 (R_free _= 0.240) for ZHX1 HD4 (OPPF_2265) and 0.203 (R_free _= 0.267) for ZHX HD2 (OPPF_2273). Statistics for the structure determinations are given in Table 4.

Assessment of any 3-D structural similarity of ZHX1 HD4 (OPPF_2265) and ZHX2 HD2 (OPPF_2273) to coordinates available in the PDB was carried with either DALI or SSM at the European Bioinformatics Institute, http://www.ebi.ac.uk/msd-srv/ssm. The ZHX homeodomain structures have been deposited in the PDB for immediate release (codes: 3NAR and 3NAU).

## Results

### ZHX construct design and protein expression

Expression of the full length ZHX genes in *E coli *was not detected, whereas division into a number of smaller constructs, yielded several soluble fragments (Table 2). Domain boundaries for these fragments relied on a combination of information from several sources. Thus we used the domain designations as shown in the NCBI protein database, as well as further bioinformatic analyses consisting of sequence alignments and consideration of low complexity regions. Where a significant region of predicted disorder was present between domains, that particular combination was not selected. Utilization of such analyses led to the design of the sixteen expression vectors for ZHX1 as outlined in Table 1. These constructs were made using Gateway technology with an overall 86% efficiency. Protein expression in *E.coli *was assessed using a robotic assay system [30], and showed that 57% gave soluble protein.

ZHX1 constructs that expressed well in small volume culture (Table 2) were scaled up and purified as shown in Table 3. Although the robotic crystallization system allowed the sampling of a wide range of conditions (8000 droplets), no crystals were obtained (Table 3). Three further complementary approaches were tried. Firstly, three selected constructs were assessed by HSQC to ascertain whether they were suitable for NMR structure determination. OPPF_1613 and OPPF_1632 had significant secondary structure (unpublished data) leading to the structure determination of the two zinc finger domain from ZHX1 [19]. Secondly, constructs for single domains of ZHX2 and ZHX3 paralogs equivalent to the ZHX1 vectors were made (Table 2), with 83% efficiency, whilst 33% of constructs gave soluble protein leading to crystals of an OPPF_2273 fragment (His-tagged ZHX2 HD2-3). No crystals were obtained for protein derived from any of the other constructs. Finally, because of the presence of a flexible polyhistidine tag, which might inhibit the packing of ZHX molecules into a crystal lattice, we explored whether tag removal would improve crystallizability. Using pOPINF, which included a rhinovirus 3C protease cleavable polyhistidine tag [22], OPPF_2264 and _2265 were purified and gave similar or enhanced yields of pure protein compared to the untagged protein (Table 3). Forty four 96-well crystallization plates were set up and crystals were obtained for OPPF_2265 (ZHX1 HD4).

### Crystal structure determination

Initial molecular replacement studies using published homeobox domain crystal structures as search models were unsuccessful. The correct molecular replacement solutions for both structures were found by using an NMR structure of the second homeobox domain of human homeodomain leucine zipper-encoding gene, homez (PDB code 1WJH, later replaced by entry 2ECC) as a search model. The search model had amino acid sequence identities of 42% to ZHX1 HD4 and respectively, 45% and 35% to the HD2 and HD3 domains of ZHX2. The structure solutions could only be obtained by deleting the flexible regions at both ends of the search molecule combined with the use of MOLREP from the CCP4 program suite. No other combination of molecular replacement program or search molecule tried provided the correct solution. The two molecules in the asymmetric unit of ZHX1 HD4 crystal are related by pseudo translation, which was detected both by MOLREP and from a native self Patterson map. After the orientation and position of the first molecule was found by MOLREP, a solution for the second molecule in the asymmetric unit was generated by applying a translation vector (0.5, 0.5, 0.5). The initial R-factor for the resolution range of 12.0-4.0Å was 0.685 which dropped to 0.517 following rigid body refinement. The extremely high initial R-factor is attributable to the fact that the two molecules have an 8° difference in orientation rather than a perfect parallel arrangement.

**Figure 2:**
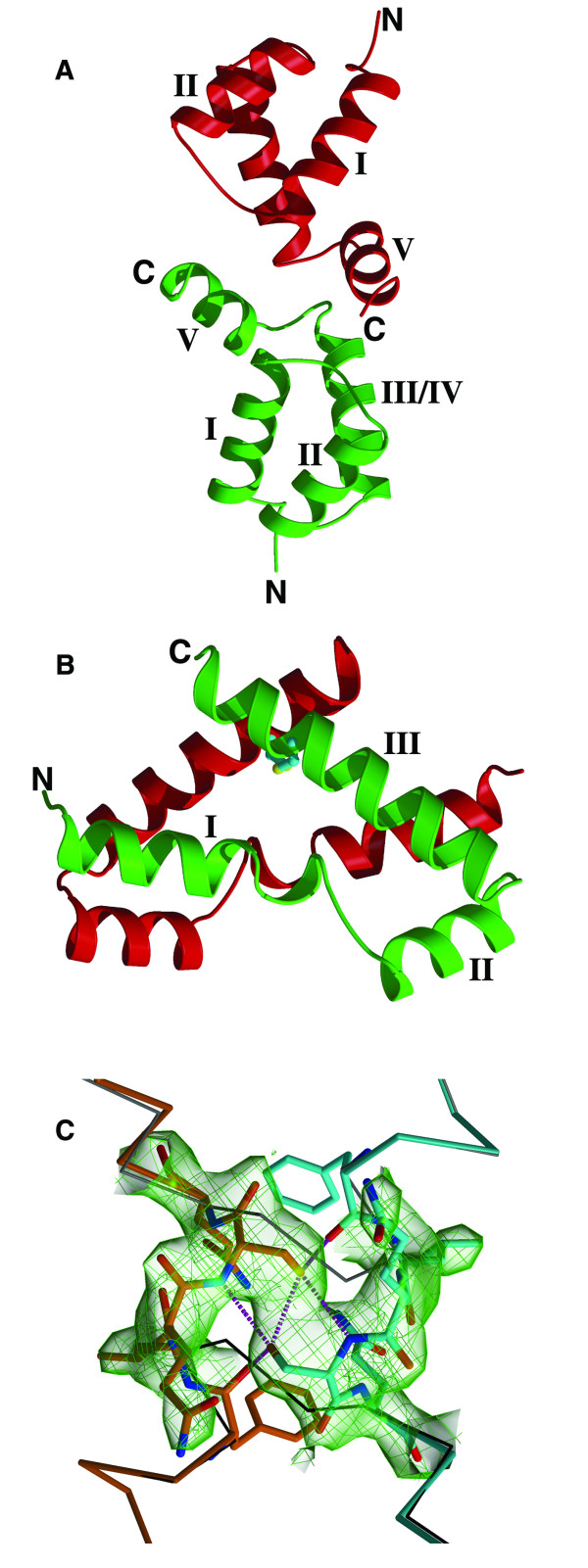
**Structures of ZHX HD domains**. (**A**) ZHX1 HD4 showing the two molecules in the crystal asymmetric unit **(B) **ZHX2 HD2 showing the two molecules in the crystal asymmetric unit. The disulfide bridge linking the subunits is shown in light blue and yellow **(C) **Part of ZHX1 HD4 showing omit electron density for residues 458-462, a region where the switch in helix I conformation occurs in ZHX2 HD2 compared to ZHX1 HD4. The monomer A is coloured in orange and monomer B in cyan. The dotted lines represent hydrogen bonds. The CA trace of a standard HD conformation is shown as thin black sticks.

The R-factor after rigid body refinement was 0.524 for all the ZHX2 HD2-3 data between the resolution range of 12.0 to 4.0 Å. It became clear, as the refinement progressed, that the two domains were identical and in fact there was a ZHX2 HD2 homodimer present in the crystal (Figure 2C). The protein had been cleaved after the crystallization droplet had been stored and the ZHX2 HD2 homodimer preferentially crystallized but in an unusual conformation, *v.i.*

### Structural analysis of ZHX1 HD4 and ZHX2 HD2

**Figure 3:**
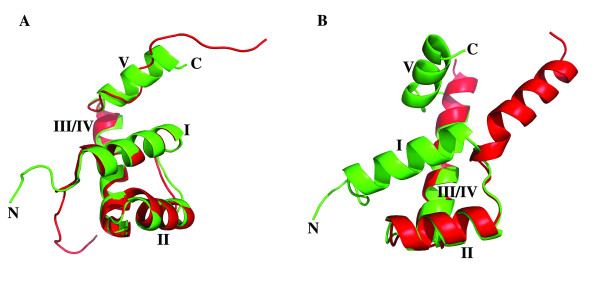
**Comparison of homeodomain structures**. **(A) **ZHX1 HD4 (green) with homez HD (2ECC, red); **(B)**. ZHX1 HD4(green) and ZHX2 HD2 (red).

**Figure 4:**
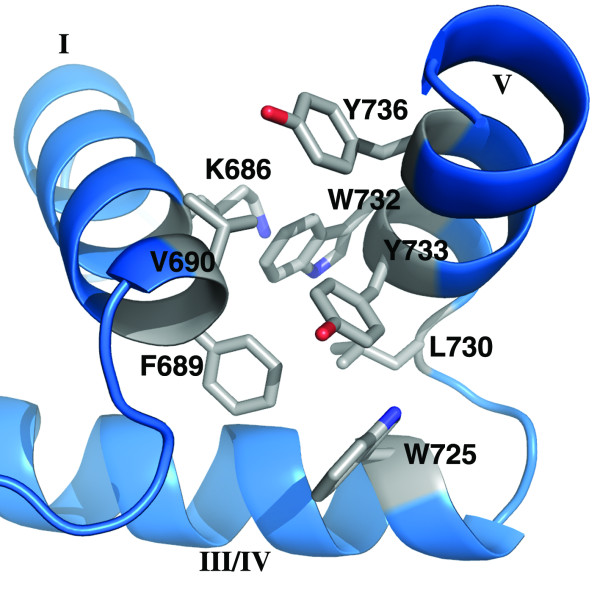
**Interactions formed by helix V in ZHX1 HD4**. Packing of ZHX1 HD4 helix V with helices I & III/IV showing extensive hydrophobic contacts involving mainly aromatic residues.

Inspection of the crystal structure of ZHX1 HD4 (OPPF_2265) showed that, in addition to the normal homeodomain secondary structure, an extra helix is located at the C-terminal region (Figure 3A, 4). The canonical homeodomain structure consists of three helices (I-III/IV) interconnected by short loops with interactions to DNA made via the N-terminal extension and helix III/IV. The additional C-terminal helix in HD4 we label as helix V in order to avoid confusion with an extension to helix III, present in certain homeodomains, referred to in earlier literature as helix IV [7]. Helices I & II are positioned anti-parallel to one another with helix III/IV located approximately normal to the plane that is formed by the N-terminal helices (Figure 2A, 3A). ZHX1 HD4 helix IV is fully ordered although no DNA is bound. Usually helix IV is disordered in homeodomain structures lacking bound DNA [31,7]. Helix V in ZHX1 HD4 is positioned almost normal to both the plane of helices I & II as well as to helix III/IV, pointing away from the DNA major groove binding site on the latter (Figure 2A, 3A). There is extensive interaction of hydrophobic side chains from helix V and particularly the C-terminal region of helix I. Many of the residues involved have aromatic side-chains including Tyr733, Trp732 and Tyr736 (Figure 4). Additional contacts are made with the C-terminal region of helix III/IV *e.g. *Trp725 with Leu730 and Tyr733.

Results from DALI revealed that the closest structural matches to ZHX1 HD4 found in the PDB were: 1), 2ECC (chainA, homeobox and leucine zipper protein, 'homez'), which had been used to solve the ZHX1 HD4 crystal structure by molecular replacement and 2), 2HDD (chain-A, transcription/DNA protein, 'engrailed' homeodomain q50k, [32])). The Z factor from DALI for homez HD2 was 10.2, with an RMS deviation of 1.5Å for 65 equivalent alpha carbons. For engrailed, the Z factor was 9.7 with an RMS deviation of 1.5Å for 55 equivalent alpha carbons. Comparison of homez (2ECC) and ZHX1 HD4 shows that the former had little more than a single turn of additional helix at the C-terminus beyond helix III/IV, although the remainder of the homez C-terminal extension partially overlaps ZHX HD4 helix V (Figure 3A). There is an overall 51% amino acid sequence identity between the homeodomain regions, whilst that beyond the initial turn of helix V is zero. Other ZHX domain structures available in the PDB *viz*: ZHX1 HD3 (PDB code 2ECB) ZHX2 HD3 (2DMP) ZHX3 HD2 (2DNO, 2DA5) appeared more dissimilar to ZHX1 HD4 than did homez or engrailed as the Z factors from DALI were all <6.8, with fewer equivalent residues found.

**Figure 5:**
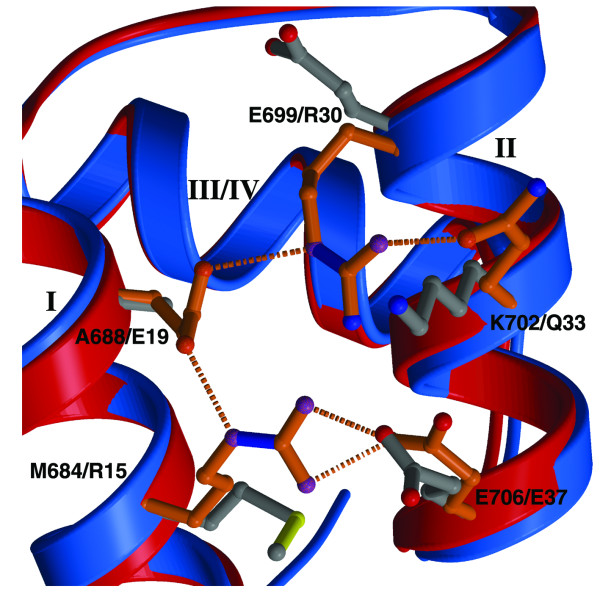
**Comparison of a region of engrailed HD and ZHX1 HD4 containing certain co-variant residues**. Engrailed HD (blue backbone, orange side-chains) showing the extensive ionic and hydrogen-bonding interactions involving commonly observed co-variant residues. These interactions are absent in ZHX1 HD4 (red backbone, gray side-chains).

Detailed comparison of ZHX HD4 with 'engrailed', revealed significant differences in the nature of the commonly observed covariant residues found in many homeodomains. Thus, in engrailed homeodomain, a network of ionic interactions is formed involving Arg15/Glu37 and Glu19/Arg30 as well as Arg15/Glu19 [33]. Such interactions have been suggested as contributing to the stabilization of the three-dimensional domain structure [31], although mutagenesis studies indicate there may be an indirect effect of the salt bridges to allow correct side-chain orientations for the optimal packing of apolar groups [34] (Figure 5). By contrast, for ZHX HD4 the network of ionic interactions is disrupted by the presence of apolar residues: Ala668 replacing Glu19 in engrailed whilst Met684 replaces Arg15. Additional hydrophobic contacts in ZHX HD4 are made via the interaction of the Met684 side-chain and the main-chain of Glu706.

The structure determination of crystals grown using protein derived from OPPF_2273 (ZHX2 HD2-3) revealed a number of unexpected features. Firstly, it became clear during the refinement that the two ZHX2 homeodomains present within the asymmetric unit were of identical amino acid sequence and were not covalently linked, unexpected for protein derived from the bidomain HD2-HD3 construct. The sequence of the observed domain corresponded to that of HD2 indicating cleavage of HD2-HD3 had occurred and that HD2 preferentially crystallized compared to HD3. This proteolysis must have largely taken place subsequent to final purification, since at the latter stage the expected size was the predominant species observed by mass spectrometry. The second unexpected feature for ZHX2 HD2 homeodomain was the unusual conformation and dimer formation. The change involved the rotation of helix I by ~180° compared to the usual homeodomain conformation, giving a more opened out structure with some disruption of the hydrophobic core. The effect of the switch on the overall conformation of an individual subunit compared to a conventional homeodomain fold can be seen in Figure 3B. A ZHX2 HD2 homodimer is observed in the crystal which is further stabilized by a disulfide bridge formed by linkage of Cys494s (Figure 2B, 3B). The switching of the positions of the two helices I in ZHX2 HD2 provides an example of 'domain swapping' [35,36].

Analysis using DALI and SSM revealed only low levels of similarity of ZHX2 HD2 to known three-dimensional protein structures, including all the available homeodomain coordinate sets. Thus, this homeodomain conformation appears to be unique amongst reported crystal/NMR structures for this domain class.

## **Discussion**

### Methodology

The bioinformatics studies, particularly the disorder prediction algorithms, were successful in identifying the additional C-terminal region that forms helix V in ZHX1 HD4. Use of sequence alignments alone would have missed this additional apparently integral feature of ZHX1 HD4, as most of helix V lacks sequence homology with other homeodomains of known structure.

Consistent with the experience from many centres using high throughput methods, our study of the ZHX family proteins has shown the value of making multiple constructs including those derived from different members of the gene family. A related approach using orthologs rather than paralogs has been recognized over many years as an important strategy in the quest to obtain diffraction quality protein crystals [37]. The variation in surface residues in differing homologs is likely to have most effect on solubility and formation of crystal contacts; being the least conserved residues they hence will vary most between different sets of orthologs or paralogs. Indeed, the homologous domains from ZHX1, 2 and 3 did not show the same pattern of expression. For example, whilst HD4 of ZHX1 expressed as a soluble protein, the equivalent domain from either ZHX2 or ZHX3 did not. Nevertheless, this work did lead to the structure for one ZHX2 homeodomain, HD2. Removal of the His tag was important for crystallizing ZHX1 HD4. Although only a small sample set was tried as a proof of principle, it may be worth pursuing tag removal if more single domain homeodomain crystal structures were to be tackled, particularly as the tag accounts for a relatively high percentage of residues in a 60 amino acid length protein

Overall the use of high-throughput cloning and expression methodology meant that many constructs could be tested, thus allowing a number of complementary approaches to be tried in order to maximize the chances of successful structure determination. These approaches led to three-dimensional structure determination for three targets (two X-ray, one NMR). Despite attempts to crystallize protein derived from multidomain constructs, only single homeodomains yielded crystals, which may imply that the linkers between domains have too much flexibility to allow packing into a lattice. Induction of rigidity in these structures, which could improve crystallisability, may require the presence of DNA oligomers or partner proteins such as additional transcription factors in higher order complexes.

### Structural

Homeodomains, with their common functional role in the regulation of gene transcription, generally show a high degree of structural conservation. As an example, certain homeodomain orthologs such as human Hox-A, although separated in evolution by 1/2 billion years from the Antennapedia gene, show >98% sequence conservation [38]. Much greater variation in sequence is, of course, observed in paralog homeodomains reflecting functional diversity, yet in spite of this, the three-dimensional structures are generally conserved. Thus the determination of two ZHX homeodomain structures reported here with variations in three-dimensional structures from the canonical form is of interest.

In ZHX1 HD4, helix IV which is in effect a C-terminal extension of helix III is well ordered, which is in contrast to the higher degree of flexibility observed in the absence of DNA [7]. The fact that ZHX1 HD4 helix IV is ordered without the bound nucleic acid ligand could be a result of the presence of helix V where its interactions with helix I may help to anchor helix IV residues. It is not clear what the functional consequences of this ordering, if any, may be. Certainly a lower level of induced fit of helix III/IV to the target DNA is likely which could in turn affect both specificity and tightness of binding of DNA.

For ZHX1 HD4, the long C-terminal helix (V) appears unusual in reported homeodomain structures. Helix V makes numerous, mainly hydrophobic contacts, with the C-terminal half of helix I. It is thus likely that this additional feature will result in greater stability of the protein. Interestingly, HD2 from homez, a homeobox leucine zipper containing transcription factor, has a short C-terminal single turn of helix that partially overlaps with helix V of ZHX1 HD4. There is no sequence identity between ZHX1 HD4 and homez homeodomain beyond this turn of helix. The extended helix V is only present in ZHX1 HD4, thus demonstrating differences in 3-D structure with homez despite their likely common origin as part of a vertebrate derived homeodomain gene subfamily [39].

Alignment of equivalent regions to ZHX1 HD4 helix V in other ZHX paralogs (Figure 1) suggests that this extended helix may also be present, particularly in the case of ZHX2. Inspection of other ZHX homeodomains whose structures have been deposited in the PDB (specifically ZHX1 HD3, ZHX2 HD3 & ZHX3 HD2), however, confirms the absence of helix V. ZHX1 HD4 has a significant amino acid sequence identity to that of homez HD2 of 51%. Homez HD2 is suggested to have a DNA binding function based on putative side-chain/nucleic acid interactions [39]. By analogy, ZHX1 HD4 has Trp25 and Arg53 in position to form hydrophobic and ionic interactions, respectively, with DNA. Additionally, the basic N-terminal region present in ZHX1 HD4 is positioned to bind to the minor groove of DNA. ZHX1 HD4 also has a significant structural relationship to an engrailed homeodomain [32,40]. Engrailed is a well characterized DNA binding homeodomain module and its structural relatedness may hence imply a similar biological role for ZHX1 HD4, rather than, for example, in formation of protein-protein interactions.

From an early study of homeodomain sequences a number of covariant residues were noted [33]. Thus 16 strongly covariant residue pairs were identified, the most highly correlated co-occurring pair of residues were Glu19 and Arg30. From the three-dimensional homeodomain structures it was clear that the side-chains of these two residues formed a salt bridge, as exemplified in the engrailed homeodomain [31] which is one of the closest related structures to ZHX1 HD4 available. For engrailed there is a network of ionic interactions formed by two sets of residues 15/37 and 19/30 which also includes 15/19. It was suggested that the salt bridges in engrailed could provide stabilization as the small core/surface ratio for such a short protein may require such additional interactions[31].

In the case of ZHX1 HD4, the ionic bridges are broken by substitution of apolar residues at 15 (residue 684 in full length ZHX1) and 19 (688). There are no other residues forming replacement salt bridges elsewhere in the ZHX HD4 structure. Homez HD2 appears different again as, although closer to ZHX HD4 in evolutionary terms, [39] it retains only a single charged side-chain in the 15/37, 19/30 set (Asp30) and as a consequence has more hydrophobic contacts in this region (*e.g. *via the side chains of Phe19 & Leu 34). Also the highly conserved 31/42 salt bridge, which is retained in ZHX1 HD4, is not present in homez where glutamine replaces aspartic acid in the former and is not even in a position to form a hydrogen bond.

Regarding the potential biological role of helix V, it appears to be unlikely to act to form a more rigid link to HD5 than those between other homeodomains in ZHX1, as there is a significant predicted region of low complexity consisting of 40 residues between HD4 and HD5. As mentioned earlier, helix V is also unlikely to directly affect DNA binding as it points away from the expected major groove binding site. Taken together with the increased hydrophobic contacts formed by covariant residues 15/37 in ZHX HD4, compared to engrailed, for example, then this could reflect the greater tendency of certain homeodomains from homeotherms to rely more on hydrophobic stabilization, as put forward in a recent pivotal study [34]. This stabilization relates to the fact that hydrophobic interactions are entropically driven and the entropy term, in contributing to the free energy decrease, has a negative temperature coefficient, thereby strengthening such interactions at higher temperature. A corollary to this is that the presence of more ionic interactions in non-homeotherm homeodomains such as engrailed may be appropriate for stabilizing the protein over a wider temperature range. Alternatively, it could be the case that lower thermal stability may be a desired property for certain transcriptional regulators, allowing a shorter protein half life and thus more rapid response as part of a control mechanism.

Naively, it might be expected that any greater stabilisation of a homeodomain could give an increased binding affinity for the DNA ligand. However, it has been observed that there is no correlation between homeodomain thermal stability and strength of DNA binding [34]. Thus the need for greater stability of an individual homeodomain may be related to specific functional requirements such as interactions with a particular set of regulatory proteins, for example, rather then representing a general property.

For OPPF_2273, the *in situ *cleavage of HD2-3 to yield crystals of ZHX2 HD2 was unexpected as was the non-canonical homeodomain conformation observed in the crystal structure involving a domain swap of helix I thereby forming a dimer. It is not clear that the manner of the generation of HD2: *i.e. *by proteolysis in the crystallization droplet, should necessarily give rise to this unusual conformation, as presumably it is merely the cleavage of a flexible loop connecting two domains. Opening out of the standard homeodomain fold, as observed for ZHX2 HD2, would likely have the effect of destabilizing the hydrophobic core of the protein. However, the dimer we observe in the crystal structure serves to bury some of the exposed hydrophobic residues at the interface between the subunits.

Previously published data indicate that HD1 of full length ZHX1-3 is responsible for both homo- and hetero-dimer formation [6,9-12], based on yeast two-hybrid data [5]. Thus the possible biological significance of a dimer formed between ZHX2 HD2 subunits in this context is unclear. As the homez/ZHX gene family is specific to a vertebrate lineage and involved in complex regulatory networks [39], then it is possible that different interactions to those detected in a yeast two-hybrid system may occur [41].

However, a similar kind of domain swapping as seen for ZHX2 HD2 could form the basis for the observed dimerisation of ZHX1 HD1 mentioned above.

Even if the unusual ZHX2 HD2 structure is not normally present at significant frequencies in biological systems it is of interest in the context of protein folding, where an alternative conformation exists for the same sequence. The altered homeodomain conformation could form the basis for the acquisition of potential distinctive new functional properties for this commonly occurring protein module.

## Conclusion

The work reported here on ZHX shows the utility of high throughput methodology for sampling a wide range of constructs. However, only single homeodomains yielded crystals in spite of soluble expression for some multi-domains. The bioinformatics methods, particularly the prediction of low complexity regions, proved useful in identifying the longer C-terminal region in ZHX HD4 which turned out to contain the novel helix V, not previously observed in other homeodomain structures. ZHX HD4 showed numerous additional hydrophobic contacts generated via interactions made by helix V. Further, the replacement of some charged covariant residues that are commonly observed to form salt bridges in non-homeotherms such as the 'engrailed' homeodomain from *Drosophila*, by non-polar side-chains further increased hydrophobic contacts within ZHX1 HD4, and therefore potentially giving greater thermostability, relative to engrailed homeodomain. ZHX1 HD4 helix V would not appear to directly affect potential DNA binding as it is orientated away from the major groove binding site on homeodomains. In contrast, for ZHX2 HD2 the rearranged conformation involving movement of helix I, relative to the canonical homeodomain fold, would disrupt the normal DNA binding site, however, protein-protein interact is possible as a homodimer is observed.

Further work will be required to explore possible roles of these ZHX homeodomains in DNA binding and dimer formation. This current study, as well as providing some new structural information on ZHX domains, has also generated a useful library of reagents for follow-up studies to determine biochemical function.

## Authors' contributions

LB designed and made all the expression vectors, did the ZHX expression testing and protein scale up work. JN carried out some ZHX purification work and did all the protein quality control tests. LB carried out the crystallizations, whilst X-ray data collection and structure determination were performed by JR. DKS conceived the project, carried out the bioinformatics studies and together with LB, JR, RJO and GF wrote the manuscript. All authors have read and approved the final manuscript.
